# Supplementary material for: Awareness and its determinant factors towards breast examination to detect breast cancer among reproductive age women in Kenya: Multi level analysis of the recent demographic and health survey data
Source: PLoS One. 2024 Dec 5;19(12):e0314700. doi: 10.1371/journal.pone.0314700 (PMC11620592; doi:10.1371/journal.pone.0314700)
Supplement: Attachment — Submitted filename: Review comments on breast examination PLOS ONE.docx [file pone.0314700.s001.docx]

**Review comments**

Thank you for the opportunity to review this paper. The title of the paper is relevant and the findings has the potential to the health of women in Kenya and Africa at large. Kindly find my comments below

**General comments**

1. Authors need to reorganize the introduction and discussion sections into meaningful paragraphs to direct the readers
2. I suggest the authors make reference to studies published in Africa more in the discussion section to support the study context.
3. Authors should improve the discussion section by stating their findings, providing reasons and recommendations but not just stating findings that otherwise support or not support their findings. This will provide further insight to the readers
4. Manuscript editing and formatting is needed to improve reading

**Abstract**

**Introduction:**

5. **“**Breast cancer is the second most frequent malignancy in women with over 2.3 million new cases and 685,000 deaths worldwide” this statement should be rephrased since it is the same as in the introduction in the main text

**Introduction**

6. Include data on prevalence of breast cancer (BC) in SSA while also citing relevant studies in Africa including such findings in Kenya.
7. Include data on the awareness level of breast examination in SSA including Kenya if any
8. Also include information of the determinants of breast examination in SSA including Kenya
9. You need to clearly articulate the problem under investigation to give the readers an opportunity to appreciate why you are conducting this study or why this study is relevant.

**Methods**

10. Include detail information about the setting of the study
11. Include detail information about the source of data. You may look at a similar study published by (Afaya et al., 2023, which used demographic and health survey data from Lesotho) for guidance.

**Design**

12. Under design, include that this is a national population-based cross-sectional survey
13. Under study design, the statement “aware of exam breast for breast cancer (s1102c)” is not clear. If possible, state this clearly.

**Variables**

**Dependent**

14. “The variable aware of exam breast for breast cancer (s1102c)” from the maternal record (IR) dataset was chosen and recoded to create the outcome variable”.

I suggest you rather include this statement in this section instead of the study design. I suggest you also state the actual question that was asked to the participants about their awareness of breast examination.

**Results**

**Demographics**

15. The statement “nearly only one tenth (11.61%) hadn’t media exposure”. Is it “had no media” exposure? Rephrase if necessary

**Discussions**

16. The statement “This could be workers would be privates and government employed recruited from more literate women” is not clear. Kindly check and rephrase for clarity
