# Supplementary material for: Awareness and its determinant factors towards breast examination to detect breast cancer among reproductive age women in Kenya: Multi level analysis of the recent demographic and health survey data
Source: PLoS One. 2024 Dec 5;19(12):e0314700. doi: 10.1371/journal.pone.0314700 (PMC11620592; doi:10.1371/journal.pone.0314700)
Supplement: Attachment — Submitted filename: Point by point response letter.docx [file pone.0314700.s002.docx]

Date: 10/07/2024

Journal: PLoS ONE

Ref: PONE-D-24-24195

Title: **Awareness and its determinant factors towards breast examination to detect breast cancer among reproductive age women in Kenya: Multi level analysis of the recent Demographic and Health Survey data**.

Authors: Mulugeta Wassie^1*^, Alebachew Ferede Zegeye^2^ ,Agazhe Aemro^2^, Bewuketu Terefe^3^, , Gebreeyesus Abera Zeleke^4^, Belayneh Shetie Workneh^5,^ Berhan Tekeba^6^, Mohamed Seid Ali^6^, Enyew Getaneh Mekonen^4^ ,Tadesse Tarik Tamir^6^

**Subject**: Submission of revised manuscript

Thank you so much for allowing us to revise our manuscript entitled “Awareness and its determinant factors towards breast examination to detect breast cancer among reproductive age women in Kenya: Multi level analysis of the recent Demographic and Health Survey data". We are very pleased for the reviewers’ and editor’s comments. We have carefully revised the manuscript and incorporated the comments accordingly. Our responses are given in point-by-point response below.

We hope the revised version is suitable for publication and look forward to hearing from you in due courses.

Sincerely

Mulugeta Wassie

Corresponding Author

**Point by point responses to Reviewers’ comments**

**Editor’s comments**

1. Please ensure that your manuscript meets PLOS ONE's style requirements, including those for file naming.

**Author’s response:** Thank you so much for your reminding. We have seen the PLOS ONE's manuscript submission style requirements carefully and tried to prepare the manuscript accordingly.

2. We noticed you have some minor occurrence of overlapping text with the following previous publication(s), which needs to be addressed: <https://www.cell.com/heliyon/fulltext/S2405-8440(20)305983?_returnURL=https%3A%2F%2Flinkinghub.elsevier.com%2Fretrieve%2Fpii%2FS2405844020305983%3Fshowall%3Dtrue>. In your revision ensure you cite all your sources (including your own works), and quote or rephrase any duplicated text outside the methods section. Further consideration is dependent on these concerns being addressed.

**Author’s response:** Thank you very much for your suggestion. We have addressed your comments and suggestions in the revised manuscript.

3. We note that your Data Availability Statement is currently as follows:

"All relevant data are within the manuscript and its Supporting Information files."

Please confirm at this time whether or not your submission contains all raw data required to replicate the results of your study. Authors must share the “minimal data set” for their submission. PLOS defines the minimal data set to consist of the data required to replicate all study findings reported in the article, as well as related metadata and methods (https://journals.plos.org/plosone/s/data-availability#loc-minimal-data-set-definition).

For example, authors should submit the following data:

- The values behind the means, standard deviations and other measures reported;

- The values used to build graphs;

- The points extracted from images for analysis.

**Author’s response:** Thank you for your comment. We have shared the dataset used to extract the results in the supporting information section of the submission system.

Additional Editor Comments:

The study examined women's awareness of breast cancer examination and associated factors in Kenya. This is very important that women have knowledge of the availability of such health services to seek care on breast cancer examination which can assist in early detection and timely treatment and surgical intervention of breast cancer. The findings from this study may have policy implications to improve care seeking behavior for breast cancer examination among women through designing and implementing evidence-based healthcare interventions at community level. The authors need to address all comments made the reviewers in order to improve the manuscript.

**Author’s Response:** We thank you for your understanding of the aim of this research. We tried to address all the comments and suggestions raised by reviewers as follows:

Review comments

**Reviewer #1:**

Thank you for the opportunity to review this paper. The title of the paper is relevant and the findings have the potential to the health of women in Kenya and Africa at large. Kindly find my comments below

General comments

1. Authors need to reorganize the introduction and discussion sections into meaningful paragraphs to direct the readers

**Authors’ response:** Thank you so much for your suggestion: We have reorganized the introduction and discussion section carefully in the revised manuscript.

2. I suggest the authors make reference to studies published in Africa more in the discussion section to support the study context.

**Authors’ response:** Thank for your suggestion. We tried to include African literatures in the discussion section although the researches related to the current title in this region are rare.

3. Authors should improve the discussion section by stating their findings, providing reasons and recommendations but not just stating findings that otherwise support or not support their findings. This will provide further insight to the readers

**Authors’ response:** Thank you for your suggestion. We have addressed it in the revised manuscript.

4. Manuscript editing and formatting is needed to improve reading

**Authors’ response:** Thank you for your suggestion and comments. We tried to proofread the whole revised manuscript in the scholarly manner.

Abstract

Introduction:

5. “Breast cancer is the second most frequent malignancy in women with over 2.3 million new cases and 685,000 deaths worldwide” this statement should be rephrased since it is the same as in the introduction in the main text

**Authors’ response:** Thank you so much for your suggestions. It is corrected in the revised manuscript.

Introduction

6. Include data on prevalence of breast cancer (BC) in SSA while also citing relevant studies in Africa including such findings in Kenya.

**Authors’ response:** Thank you so much for your comment. It has been included in the revised manuscript.

7. Include data on the awareness level of breast examination in SSA including Kenya if any

**Authors’ response:** Thank you for your comment. Although there are limited literatures, we tried to address them in the revised manuscript.

8. Also include information of the determinants of breast examination in SSA including Kenya

**Authors’ response:** Dear reviewer, thank you for your comment. The comments have been addressed in the revised manuscript.

9. You need to clearly articulate the problem under investigation to give the readers an opportunity to appreciate why you are conducting this study or why this study is relevant.

**Authors’ response:** Dear reviewer, thank you for your comment. The authors have tried to include this comment in the revised manuscript.

Methods

10. Include detail information about the setting of the study

**Authors’ response:** Thank you for your suggestions. We have included detailed information about the setting in the revised manuscript.

11. Include detail information about the source of data. You may look at a similar study published by (Afaya et al., 2023, which used demographic and health survey data from Lesotho) for guidance.

**Authors’ response:** Thank you so much for your comments and suggestions. We have included it in the revised manuscript.

Design

12. Under design, include that this is a national population-based cross-sectional survey

**Authors’ response:** Thank you so much for your comments. It is included in the revised manuscript.

13. Under study design, the statement “aware of exam breast for breast cancer (s1102c)” is not clear. If possible, state this clearly.

**Authors’ response:** Thank you for your suggestion. It is directly taken as it appears in the DHS dataset. It is rephrased in the revised manuscript.

Variables

Dependent

14. “The variable aware of exam breast for breast cancer (s1102c)” from the maternal record (IR) dataset was chosen and recoded to create the outcome variable”.

I suggest you rather include this statement in this section instead of the study design. I suggest you also state the actual question that was asked to the participants about their awareness of breast examination.

**Authors’ response:** Thank you for your suggestion. We put it on this section and it is rephrased as “are you aware of breast exam to detect breast cancer”.

Results

Demographics

15. The statement “nearly only one tenth (11.61%) hadn’t media exposure”. Is it “had no media” exposure? Rephrase if necessary

**Authors’ response:** Thank you for your suggestion. We have rephrased it in the revised manuscript.

Discussions

16. The statement “This could be workers would be privates and government employed recruited from more literate women” is not clear. Kindly check and rephrase for clarity.

**Authors’ response:** Thank you for your suggestion. We have seen it and rephrased in the revised manuscript.

**Reviewer #2:**

Thank you for giving me the opportunity to review this important topic. Here are my comments.

Strengths:

1. Relevant and important topic

2. Large and updated data set

Weaknesses: The authors should address the following comments before acceptance.

Abstract:

1- Results: The authors should add AOR with 95%CI for significant variables.

**Authors’ response:** Dear reviewer, thank you so much for your comments.

We have included all these information in the revised manuscript.

2- Conclusion: Please be specific and policy relevant.

**Authors’ response:** thank you so much for your suggestion. We have tried to revise it in the revised manuscript.

Introduction:

- The introduction needs to be comprehensively revises. The authors should add:

- The significance of breast examination in breast cancer detections.

- What was the level and determinants in earlier studies conducted in Kenya, If there are no studies the authors should focus on other LMICs.

- What is the current national policy? Are there any interventions for improving awareness in Kenya now?

- The authors can also mention on the importance of research.

**Authors’ response:** thank you so much for your suggestion. We have addressed all the issues raised in the revised manuscript.

Methods:

-The authors should mention what variables were selected in MLR.

**Authors’ response:** thank you so much for your comments. It is addressed in the “Data processing and statistical analysis” section of the revised manuscript.

Results:

- Please write the results of MLR analysis in correct format: [AOR, 95%CI:]. The format used is not similar for all variables.

**Authors’ response:** thank you so much for your comments. It is addressed in the revised manuscript.

Discussion:

This needs a bit more work. At current form it is a repetition of the results. A good discussion should have four arms: 1) present key finding, 2) compare with other literature, 3) rational and 4) policy implication. I suggest you revise your discussion based on the above criteria. Present each key finding, compare with earlier literature, and provide policy and practical implications for them. Each finding should have a key implication for practice and policy.

**Authors’ response:** thank you so much for your comments. We have tried to revise the discussion based on the comments in the revised manuscript.

Finally, I would highly recommend that you guys have a native English speaker (someone who grew up in an environment with English as their mother tongue) to review the readability of this article. I do think that you guys have decent English, but it comes across as janky and the flow is quite uneven. Being a non native English speaker I understand the struggle you guys are facing. Unfortunately, there is a limit to how much we can do on our own. It is imperative that we get our articles proofread by a native English speaker (preferably someone from America to ensure wider acceptance). Therefore, I strongly recommend improving the language of the article.

**Authors’ response:** Dear reviewer, we really appreciate your deep understanding. Even though still there could be some unseen typos, we tried to improve the language of the revised manuscript.
