# Supplementary material for: Awareness and its determinant factors towards breast examination to detect breast cancer among reproductive age women in Kenya: Multi level analysis of the recent demographic and health survey data
Source: PLoS One. 2024 Dec 5;19(12):e0314700. doi: 10.1371/journal.pone.0314700 (PMC11620592; doi:10.1371/journal.pone.0314700)
Supplement: Attachment — Submitted filename: PLOS one second review.docx [file pone.0314700.s003.docx]

Dear Editor,

Thank you for allowing me to review this manuscript for the second time. I appreciate your patience in waiting for my response. Generally, the authors have addressed all my comments and I give them a thumbs up for that. Find some minor comments for the authors.

Comments to authors.

I see that the discussion section has a lot of disjointed paragraphs. These paragraphs could be well-aligned or merged to improve the reading and flow of the message. Statements that are talking about the same thing should be put together. Example;

“The result differences might be due to the sample size differences, as the current study is a national-based survey, in addition to the sociocultural differences of the given countries. The current result could be an alarming sign for policymakers and other stakeholders to implement socio-culturally acceptable ways of awareness-creation modalities to improve breast examinations to detect breast cancer in the country”.

This paragraph should be added to the first to make it a complete sentence
