# Supplementary material for: Awareness and its determinant factors towards breast examination to detect breast cancer among reproductive age women in Kenya: Multi level analysis of the recent demographic and health survey data
Source: PLoS One. 2024 Dec 5;19(12):e0314700. doi: 10.1371/journal.pone.0314700 (PMC11620592; doi:10.1371/journal.pone.0314700)
Supplement: Attachment — Submitted filename: Response to Reviewers.docx [file pone.0314700.s004.docx]

Date: 15/10/2024

Journal: PLoS ONE

Ref: PONE-D-24-24195R1

Title: **Awareness and its determinant factors towards breast examination to detect breast cancer among reproductive age women in Kenya: Multi level analysis of the recent Demographic and Health Survey data**.

Authors: Mulugeta Wassie^1*^, Alebachew Ferede Zegeye^2^ ,Agazhe Aemro^2^, Bewuketu Terefe^3^, , Gebreeyesus Abera Zeleke^4^, Belayneh Shetie Workneh^5,^ Berhan Tekeba^6^, Mohamed Seid Ali^6^, Enyew Getaneh Mekonen^4^ ,Tadesse Tarik Tamir^6^

**Subject**: Submission of 2nd revised manuscript

Thank you so much for allowing us to revise our manuscript entitled “Awareness and its determinant factors towards breast examination to detect breast cancer among reproductive age women in Kenya: Multi level analysis of the recent Demographic and Health Survey data". We are very pleased for the reviewers’ and editor’s comments. We have carefully revised the manuscript and incorporated the comments accordingly. Our responses are given in point-by-point response below.

We hope the revised version is suitable for publication and look forward to hearing from you in due courses.

Sincerely

Mulugeta Wassie

Corresponding Author

**Point by point responses to Reviewers’ comments**

**Reviewer #1:**

I see that the discussion section has a lot of disjointed paragraphs. These paragraphs could be well-aligned or merged to improve the reading and flow of the message. Statements that are talking about the same thing should be put together. Example;

“The result differences might be due to the sample size differences, as the current study is a national-based survey, in addition to the sociocultural differences of the given countries. The current result could be an alarming sign for policymakers and other stakeholders to implement socio-culturally acceptable ways of awareness-creation modalities to improve breast examinations to detect breast cancer in the country”.

This paragraph should be added to the first to make it a complete sentence

**Authors’ response:** Thank you so much for your constructive comments. All the concerns are addressed in the revised manuscript.

**Reviewer #2:**

Dear Authors,

The authors have made several changes that were recommended in the initial review, and the current submission is a much more relevant and interesting article to read. The added discussion of factors that make the women of Kenya uniquely susceptible adds context and relevance to the study. There are still many points that need to be addressed before this manuscript can be accepted for publication.

**Major Points:**

Introduction: The Introduction is still an issue. As the first part of the article that a potential reader reads, it should be attractive and easy to read. You have to "sell" the article to the reader.

**Authors’ response:** Thank you so much for your unreserved effort and your comments. We tried to incorporate the comments in the main manuscript.

Results: The statistical methods employed should be explained in methods section.

**Authors’ response:** Thank you so much for your suggestion. The statistical methods used in the results section are explained at the data processing and analysis section (from lines 115 to 124) and Random effects and model fitness section (from lines 129 to 138).

Discussion: Discussion needs more work. Based on the previous comments, the authors should present key findings compare with other studies, possible explanations, and key practical implications?

**Authors’ response**: Thank you for your constructive comments. We tried to address the comments in the revised manuscript.

**Minor points:**

Abstract:

- Methods: A total of 16,474 women were included. Please revise as: A total of 16,474 women of reproductive age were included.

**Authors’ response:** Thank you for your comment. It is corrected as per the suggestion given.

- Results: Please follow correct spacing. The authors should give a space after comma. There are a lot of spacing and punctuation errors throughout the manuscript.

**Authors’ response**: Thank you for your comment. We tried to address the comments in the main revised document.

- Conclusion: The authors’ recommend- Please revise as the study recommends. Please use small letter for Media.

Authors’ response: Thank you for your recommendation: It is addressed in the revised document.

Introduction:

- Please give a space between citation and preceding word. Line: 6: Respectively(2,3). It should be respectively (2,3). Please follow this in whole manuscript. Use your citations as per journal guideline [2,3].

**Authors’ response:** Thank you for your suggestion. We tried to incorporate the comments in the revised manuscript.

Methods:

- Study variables: Please remove the variable number and the data set number.

**Authors’ response:** Thank you for your suggestion. We have corrected it in the revised manuscript.

Results:

- weren't married: Were not married

- hadn't any information: had no information

- Please remove dot before . (Table 1).

Table 1. Please use one number after coma (18.93%, make it 18.9%). Please revise all table.

Table 1. What does non married mean?

Table 2. Please two numbers after coma. For example: 1.156 (1.014-1.318). It should be 1.15 (1.01-1.31). Please revise all table.

**Authors’ response:** We have corrected the comments as per your suggestion in the result section of the manuscript. “Non married” in the table one was to mean single mam. In case it made confusion, we changed it to “single” in the revised manuscript.

This manuscript need major editing for language and grammar. At its current form, the language of the article is not appropriate for academic publishing.

**Authors’ response:** Thank you for your constructive comments to make the manuscript more scientific. We tried to solve the grammatical issue of the manuscript in the revised version.

In the revised manuscript, please give page and line numbers (using continuous options). The authors also have to revise their data availability statement and how can be data accessed. I believe the data will have a repository link.

**Authors’ response:** Thank you for your constructive comments. We have given the continuous line number in the revised manuscript. The data availability statement is also revised.
